# Leukocyte telomere length and diabetes status, duration, and control: the 1999–2002 National Health and Nutrition Examination Survey

**DOI:** 10.1186/s12902-015-0050-1

**Published:** 2015-09-29

**Authors:** Andy Menke, Sarah Casagrande, Catherine C. Cowie

**Affiliations:** Social & Scientific Systems, Inc, 8757 Georgia Avenue, 12th Floor, Silver Spring, MD 20910 USA; National Institute of Diabetes and Digestive and Kidney Diseases, 6707 Democracy Blvd, Rm 691, MSC 5460, Bethesda, MD 20892 USA

**Keywords:** Telomere, Diabetes mellitus, Aging

## Abstract

**Background:**

Studies investigating the association between telomere length and diabetes have been inconsistent, and there are few data available investigating the associations of telomere length with diabetes duration and control. We evaluated the relationship of leukocyte telomere length with diabetes, and the relationship of leukocyte telomere length with diabetes duration and poor glucose control among people with diabetes.

**Methods:**

We used data from the 1999–2002 National Health and Nutrition Examination Survey, a representative sample of the US civilian non-institutionalized population. In 3921 participants, leukocyte telomere length was measured and diabetes status was determined based on a previous diagnosis, hemoglobin A1c ≥6.5 %, or fasting glucose ≥126 mg/dL.

**Results:**

The odds ratios (95 % confidence intervals) of diabetes associated with the first, second, and third quartile of leukocyte telomere length, compared to the highest quartile, was 2.09 (1.46–2.98), 1.74 (1.30–2.31), and 1.08 (0.76–1.54), respectively (*p*-trend < 0.01), in unadjusted models and 0.74 (0.48–1.14), 0.91 (0.61–1.34), and 0.87 (0.59–1.29), respectively (*p*-trend = 0.20), in multivariable adjusted models. Among participants with diabetes, unadjusted and adjusted leukocyte telomere length was not associated with diabetes duration or glucose control based on an hemoglobin A1c <7 or <8 % (all *p* > 0.05).

**Conclusions:**

In this study of the US general population, leukocyte telomere length was not associated with diabetes status, diabetes duration, or diabetes control.

## Background

Telomeres are DNA protein structures at the end of all chromosomes consisting of tandem repeats of TTAGGG sequence [1]. They preserve the chromosomes’ stability and integrity by overcoming the problem of DNA polymerase’s inability to fully copy the very end of the lagging strand. The resulting telomere shortening is a sign of physiologic aging and eventually leads to cell senescence. A faster rate of attrition and consequently premature cell senescence may be a cause or a consequence of type 2 diabetes [2]. Shorter telomeres leading to senescence and apoptosis of the beta cells may contribute to more rapid development of type 2 diabetes. Also, diabetes and obesity are associated with increased oxidative stress, which may in turn lead to telomere shortening [2, 3]. Several previous studies have investigated the association between leukocyte telomere length (LTL) and diabetes with inconsistent results [4–10]. This association has not been studied in a representative sample of the US general population. Furthermore, few data are available investigating the association of LTL with diabetes duration or control [7].

We evaluated the relationship of LTL and diabetes using data from the 1999–2002 National Health and Nutrition Examination Surveys (NHANES), a representative sample of the US civilian non-institutionalized population. In addition, we evaluated the relationship between LTL and poor glucose control and diabetes duration among people with diabetes.

## Methods

### Study population

We limited our analysis to the years 1999–2002 because those were the only years NHANES measured LTL. NHANES 1999–2002 is a stratified, multistage probability survey designed to be representative of the civilian, non-institutionalized US population [11]. The de-identified datasets are publicly available at http://www.cdc.gov/nchs/nhanes/nhanes_questionnaires.htm. For the examination portion of the survey, participants were randomly allocated to either a morning examination for which they are asked to fast (8 to <24 h) or an afternoon/evening examination. We used data from the morning examination session in order to include fasting glucose in the definition of diabetes; the morning examination data is capable of independently producing national estimates. Of 4798 adults ≥20 years of age, we excluded three participants with likely type 1 diabetes (diagnosis <30 years of age and initiation of insulin within 1 year of diagnosis), 233 pregnant women, and 641 participants missing telomere data, resulting in a final sample of 3921 participants.

The protocol for the 1999–2002 NHANES was approved by the National Center for Health Statistics (NCHS) of the Centers for Disease Control and Prevention research ethics board. All participants gave written informed consent.

### Data collection

Data for NHANES 1999–2002 were collected during an in-home interview and a subsequent visit to a mobile examination center (MEC) [11]. Standardized questionnaires were used to collect information regarding age, race-ethnicity, sex, education, household income, smoking status, pack years smoked, and alcohol consumption during the in-home interview. During the examination, height and weight were measured and body mass index (BMI; weight [kg]/height [m^2^]) was calculated. A trained phlebotomist obtained a blood sample according to a standardized protocol and serum C-reactive protein (CRP) was measured using latex-enhanced nephelometry, a high sensitivity assay.

Participants self-reported a previous diagnosis of diabetes by a doctor or other health professional. Hemoglobin A1c (A1c), a measure of average glucose levels over the past three months, was measured in whole blood using a Primus Automated HPLC system (Primus Corp., Kansas City, MO) and the interassay coefficient of variation ranged from 1.1 to 2.0 %. Fasting plasma glucose was measured using a Roche Cobas Mira Chemistry System (Roche Diagnostics Systems, Inc., Montclair, New Jersey) and the interassay coefficient of variation ranged from 1.3 to 3.0 %. We defined diabetes as a previous diagnosis of diabetes, A1c ≥6.5 % (48 mmol/mol), or fasting glucose ≥126 mg/dL [12].

Aliquots of DNA purified from whole blood were provided by the Division of Health and Nutrition Examination Surveys, National Center for Health Statistics, Centers for Disease Control and Prevention [13]. The laboratory of Elizabeth Blackburn at the University of California, San Francisco performed the LTL assay using the quantitative polymerase chain reaction method to measure LTL relative to a reference DNA (T/S ratio) [14, 15]. Each sample was assayed twice and if the variability was >7 %, then a third assay was run. The two closest T/S ratio values were used to calculate an average. The interassay coefficient of variation was 4.4 %.

### Statistical methods

LTL were categorized into quartiles based on the weighted sample distribution of LTL. We calculated means (standard errors) and percentages (standard errors) of participant characteristics by quartile of LTL. We used logistic regression to estimate the odds ratios and 95 % confidence intervals of diabetes associated with each quartile of LTL. We tested for linear trends across quartiles of LTL by including the median LTL value for each quartile as a continuous variable in logistic regression models. Initial regression models were unadjusted; a subsequent model was adjusted for age, race-ethnicity, and sex, while a final multivariable model adjusted for age, race-ethnicity, sex, education, income, smoking, pack years smoked, alcohol consumption, body mass index, and C-reactive protein.

We also calculated the geometric mean LTL by diabetes status (normal glucose level/prediabetes/diabetes), duration of diabetes among people with diabetes (undiagnosed/diagnosed with duration < 3 years/3–5 years/6–10 years/≥11 years), and A1c control among people with diagnosed diabetes (using 7 % [53 mmol/mol] and 8 % [64 mmol/mol] cutpoints based on American Diabetes Association treatment goals) [16]. To do so, we calculated conditional margins (essentially adjusted mean values of the dependent variable for each category of an independent variable) [17] from linear regression models with LTL modeled as a continuous, log-transformed variable using adjustment identical to the models described above. *P*-values for differences in geometric mean LTL by diabetes status, duration, and control were calculated using Wald F-tests.

Appropriate sample weights were used in all analyses so that the sum of the sample weights (MEC and fasting weights) added to the total civilian non-institutionalized US population; weights were used to account for unequal probabilities of selection and non-response and thus provide estimates representative of the non-institutionalized US population. Data analyses were done using SUDAAN (version 10.0.1; RTI International, Research Triangle Park, NC) accounting for the stratified, clustered sample design used by NHANES.

## Results

Participants in lower quartiles of LTL were older, more likely to be non-Hispanic white, less likely to be non-Hispanic black, and less likely to be women compared to their counterparts in higher LTL quartiles (*p*-trend <0.05 for all; Table 1). They were less likely to have a high school education or to be never smokers; they had higher mean pack years smoked (among smokers), body mass index, and A1c levels; and they were more likely to have C-reactive protein ≥1.0 mg/dL and diabetes (*p*-trend <0.05 for all).

**Table 1:** Characteristics^a^ of participants by quartile of leukocyte telomere length^b^, 1999–2002

	LTL	LTL	LTL	LTL	
	<0.87	0.87–1.01	1.02–1.19	≥1.20	*p*-trend
Age, years	55.4 (0.7)	48.5 (1.1)	42.4 (1.0)	37.9 (1.0)	<0.01
Non-Hispanic white, %	77.9 (3.1)	75.9 (2.5)	72.7 (2.9)	66.7 (2.9)	<0.01
Non-Hispanic black, %	7.7 (1.2)	8.6 (1.6)	8.7 (1.3)	13.2 (2.3)	0.01
Mexican-American, %	6.0 (1.3)	7.1 (1.2)	7.9 (1.2)	6.5 (1.2)	0.76
Women, %	45.3 (1.6)	53.0 (2.1)	51.2 (2.3)	51.7 (2.1)	0.04
<High school education, %	26.7 (1.4)	21.4 (1.5)	19.9 (1.8)	18.0 (1.6)	<0.01
High school education, %	25.3 (1.9)	28.9 (2.5)	25.6 (2.3)	27.6 (2.4)	0.67
>High school education, %	47.9 (2.0)	49.7 (2.6)	54.4 (2.7)	54.4 (2.7)	0.03
Household income < $20,000, %	26.5 (1.7)	21.2 (1.6)	21.9 (2.3)	26.8 (3.0)	0.81
Current smokers, %	22.1 (1.6)	26.1 (2.3)	24.6 (2.3)	26.6 (2.4)	0.22
Former smokers, %	32.9 (1.7)	28.6 (2.4)	23.4 (1.8)	21.1 (1.9)	<0.01
Never smokers, %	45.1 (2.0)	45.4 (1.8)	52.0 (2.4)	52.3 (3.1)	<0.01
Pack years smoked, mean years	29.5 (1.6)	25.2 (1.1)	19.5 (1.1)	14.5 (1.2)	<0.01
Consume alcohol, %	68.9 (1.6)	73.7 (2.9)	74.1 (3.1)	76.0 (3.4)	0.10
Body mass index, kg/m^2^	28.6 (0.3)	28.4 (0.3)	27.6 (0.3)	27.4 (0.3)	<0.01
C-reactive protein ≥1 mg/dL, %	12.9 (1.3)	10.1 (1.0)	7.8 (1.0)	8.7 (1.3)	0.04
Diabetes, %	12.9 (0.8)	11.0 (0.9)	7.1 (0.8)	6.6 (0.8)	<0.01
A1c, mean %	5.6 (0.03)	5.5 (0.03)	5.4 (0.03)	5.3 (0.03)	<0.01
A1c, mean mmol/mol	34 (0.3)	37 (0.3)	36 (0.3)	34 (0.3)	<0.01

*LTL* leukocyte telomere length

^a^Mean (standard error) or percentage (standard error)

^b^Telomere length was measured relative to standard reference DNA and presented as a ratio (T/S ratio)

The unadjusted odds ratios and 95 % confidence intervals (95 % CI) of diabetes associated with the first, second, and third quartile of LTL, compared to the highest quartile, was 2.09 (95 % CI, 1.46–2.98), 1.74 (95 % CI, 1.30–2.31), and 1.08 (95 % CI, 0.76–1.54), respectively (*p*-trend < 0.01; Table 2). After multivariable adjustment, the analogous estimates were 0.74 (95 % CI, 0.48–1.14), 0.91 (95 % CI, 0.61–1.34), and 0.87 (95 % CI, 0.59–1.29), respectively (*p*-trend = 0.20).

**Table 2:** Odds ratios (95 % confidence intervals) of diabetes associated with quartile of leukocyte telomere length^a^, 1999–2002

	LTL	LTL	LTL	LTL	
	<0.87	0.87–1.01	1.02–1.19	≥1.20	*p*-trend
Unadjusted	2.09 (1.46–2.98)	1.74 (1.30–2.31)	1.08 (0.76–1.54)	1.00	<0.01
Age, race-ethnicity, and sex adjusted	0.89 (0.63–1.26)	1.08 (0.80–1.47)	0.86 (0.62–1.19)	1.00	0.73
Multivariable adjusted^b^	0.74 (0.48–1.14)	0.91 (0.61–1.34)	0.87 (0.59–1.29)	1.00	0.20

*LTL* leukocyte telomere length

^a^Telomere length was measured relative to standard reference DNA and presented as a ratio (T/S ratio)

^b^Multivariable model adjusted for age, race-ethnicity, sex, education, income, smoking, pack years smoked, alcohol consumption, body mass index, and C-reactive protein

Geometric mean LTL was not associated with diabetes status, duration of diabetes, or control of diabetes after age, race-ethnicity, and sex adjustment or multivariable adjustment (Table 3). In multivariable adjusted models, the geometric mean LTL was 1.000 (95 % CI, 0.967–1.034) for normal glucose levels, 1.009 (95 % CI, 0.970–1.050) for prediabetes, and 1.032 (95 % CI, 0.996–1.069) for diabetes (*p* = 0.11). Among participants with diabetes, the multivariable adjusted geometric mean LTL was 0.957 (95 % CI, 0.921–0.994) for undiagnosed diabetes, 0.983 (95 % CI, 0.917–1.055) for 0–2 years duration, 0.971 (95 % CI, 0.909–1.037) for 3–5 years duration, 0.974 (95 % CI, 0.904–1.050) for 6–10 years duration, and 0.989 (95 % CI, 0.940–1.041) for ≥11 years duration (*p* = 0.57). Among participants with diagnosed diabetes, the multivariable geometric mean LTL was 0.955 (95 % CI, 0.911–1.001) for A1c <7 % and 0.939 (95 % CI, 0.904–0.976) for A1c ≥7 % (*p* = 0.43).

**Table 3:** Geometric mean (95 % confidence interval) leukocyte telomere length^a^ by diabetes status, diabetes control, and diabetes duration, 1999–2002

	Unadjusted	Age, race-ethnicity, and sex adjusted	Multivariable adjusted^b^
Diabetes status	*p* < 0.01	*p* = 0.68	*p* = 0.11
Normal	1.045 (1.010–1.082)	1.012 (0.979–1.046)	1.000 (0.967–1.034)
Prediabetes	0.970 (0.937–1.006)	1.010 (0.974–1.048)	1.009 (0.970–1.050)
Diabetes	0.946 (0.918–0.974)	1.020 (0.989–1.053)	1.032 (0.996–1.069)
Duration among those with diabetes	*p* = 0.44	*p* = 0.90	*p* = 0.57
Undiagnosed	0.970 (0.937–1.006)	0.965 (0.932–0.999)	0.957 (0.921–0.994)
Duration 0–2 years	0.977 (0.916–1.041)	0.966 (0.901–1.035)	0.983 (0.917–1.055)
Duration 3–5 years	0.969 (0.920–1.022)	0.984 (0.938–1.032)	0.971 (0.909–1.037)
Duration 6–10 years	0.950 (0.889–1.015)	0.974 (0.918–1.033)	0.974 (0.904–1.050)
Duration ≥11 years	0.927 (0.884–0.972)	0.982 (0.938–1.028)	0.989 (0.940–1.041)
Control among diagnosed diabetes (7 %)	*p* = 0.43	*p* = 0.65	*p* = 0.43
A1c <7 % (53 mmol/mol)	0.943 (0.909–0.978)	0.954 (0.918–0.992)	0.955 (0.911–1.001)
A1c ≥7 % (53 mmol/mol)	0.956 (0.920–0.993)	0.947 (0.913–0.982)	0.939 (0.904–0.976)
Control among diagnosed diabetes (8 %)	*p* = 0.80	*p* = 0.12	*p* = 0.20
A1c <8 % (64 mmol/mol)	0.948 (0.915–0.983)	0.961 (0.927–0.997)	0.957 (0.918–0.999)
A1c ≥8 % (64 mmol/mol)	0.953 (0.912–0.997)	0.930 (0.891–0.971)	0.926 (0.882–0.973)

^a^Telomere length was measured relative to standard reference DNA and presented as a ratio (T/S ratio)

^b^Multivariable model adjusted for age, race-ethnicity, sex, education, income, smoking, pack years smoked, alcohol consumption, body mass index, and C-reactive protein

## Discussion

In this large, population based study of the US general population, lower LTL was associated with higher prevalence of diabetes in unadjusted models. However, in models adjusted for age, race-ethnicity, and sex and in multivariable adjusted models, the association between LTL and diabetes was attenuated and no longer significant. In addition, LTL was not associated with diabetes duration or diabetes control in unadjusted or adjusted models.

Previous studies investigating the association between diabetes status and LTL have been inconsistent. In a cross-sectional study and in three case–control studies, participants with diabetes had shorter LTL than those without diabetes [5–7, 9]. In the Strong Heart Study, a prospective cohort study of participants without diabetes at baseline, those in the lowest quartile of LTL had an 83 % higher risk of developing diabetes after 5.5 years of follow-up than those in the highest quartile of LTL [10]. Among participants of the Finnish Diabetes Prevention Study with impaired glucose tolerance, baseline LTL was not associated with the development of diabetes after 8.5 years of follow-up [4]. In a nested case–control study including postmenopausal women participating in the Women’s Health Initiative, baseline LTL were similar among women who developed diabetes and those who did not develop diabetes after 6 years of follow-up; this study also found no association between telomere length and diabetes risk in a Mendelian randomization analysis [8].

Since diabetes and obesity are associated with greater oxidative stress, the conditions may also cause more rapid LTL shortening [2], and consequently those with a longer diabetes duration and poor glucose control may experience faster LTL attrition. However, we found no evidence of an association in our study. We were only able to identify one previous study investigating the association between duration of diabetes and LTL, and it found no association [7]. Among people with diabetes, LTL may be related to complications, as previous studies in diabetes populations have found that LTL was associated with renal disease [18, 19].

Our study had a number of strengths, including use of a rigorous study protocol with extensive quality control procedures and technicians trained and certified in all data collection procedures. The sample size was large with a sufficient number of diabetic participants to investigate the associations of diabetes duration and control with LTL. Also, the results of NHANES are generalizable to the civilian, noninstitutionalized US population. However, the study had limitations, including a cross-sectional study design with LTL measured from a single DNA specimen; therefore, we did not have data on the rate of LTL shortening. Telomere length may vary among cells in the same tissue and among chromosomes in the same cell, but a previous study showed telomere length from different tissues within the same person are highly correlated [20].

## Conclusions

In conclusion, we did not find an association between LTL and diabetes status, duration, or control after adjustment. Our results suggest telomere attrition is not a cause or a consequence of diabetes.

## Abbreviations

A1c: Hemoglobin A1c
CRP: C-reactive protein
LTL: Leukocyte telomere length
MEC: Mobile examination center
NCHS: National Center for Health Statistics
NHANES: National Health and Nutrition Examination Survey

## References

[1] Riethman H (2008). Human telomere structure and biology. Annu Rev Genomics Hum Genet.

[2] Sampson MJ, Hughes DA (2006). Chromosomal telomere attrition as a mechanism for the increased risk of epithelial cancers and senescent phenotypes in type 2 diabetes. Diabetologia.

[3] von Zglinicki T (2002). Oxidative stress shortens telomeres. Trends Biochem Sci.

[4] Hovatta I, de Mello VD, Kananen L, Lindstrom J, Eriksson JG, Ilanne-Parikka P (2012). Leukocyte telomere length in the Finnish Diabetes Prevention Study. PLoS One.

[5] Olivieri F, Lorenzi M, Antonicelli R, Testa R, Sirolla C, Cardelli M (2009). Leukocyte telomere shortening in elderly Type2DM patients with previous myocardial infarction. Atherosclerosis.

[6] Salpea KD, Talmud PJ, Cooper JA, Maubaret CG, Stephens JW, Abelak K (2010). Association of telomere length with type 2 diabetes, oxidative stress and UCP2 gene variation. Atherosclerosis.

[7] Shen Q, Zhao X, Yu L, Zhang Z, Zhou D, Kan M (2012). Association of leukocyte telomere length with type 2 diabetes in mainland Chinese populations. J Clin Endocrinol Metab.

[8] You NC, Chen BH, Song Y, Lu X, Chen Y, Manson JE (2012). A prospective study of leukocyte telomere length and risk of type 2 diabetes in postmenopausal women. Diabetes.

[9] Zee RY, Castonguay AJ, Barton NS, Germer S, Martin M (2010). Mean leukocyte telomere length shortening and type 2 diabetes mellitus: a case–control study. J Lab Clin Med.

[10] Zhao J, Zhu Y, Lin J, Matsuguchi T, Blackburn E, Zhang Y (2014). Short leukocyte telomere length predicts risk of diabetes in american indians: the strong heart family study. Diabetes.

[11] Zipf G, Chiappa M, Porter KS, Ostchega Y, Lewis BG, Dostal J (2013). National Health and Nutrition Examination Survey: plan and operations, 1999–2010. National Center for Health Statistics. Vital Health Stat.

[12] American Diabetes Association (2010). Diagnosis and classification of diabetes mellitus. Diabetes Care.

[13] Needham BL, Adler N, Gregorich S, Rehkopf D, Lin J, Blackburn EH (2013). Socioeconomic status, health behavior, and leukocyte telomere length in the National Health and Nutrition Examination Survey, 1999–2002. Soc Sci Med.

[14] Cawthon RM (2002). Telomere measurement by quantitative PCR. Nucleic Acids Res.

[15] Lin J, Epel E, Cheon J, Kroenke C, Sinclair E, Bigos M (2010). Analyses and comparisons of telomerase activity and telomere length in human T and B cells: insights for epidemiology of telomere maintenance. J Immunol Methods.

[16] American Diabetes Association (2013). Diabetes Care.

[17] Graubard BI, Korn EL (1999). Predictive margins with survey data. Biometrics.

[18] Tentolouris N, Nzietchueng R, Cattan V, Poitevin G, Lacolley P, Papazafiropoulou A (2007). White blood cells telomere length is shorter in males with type 2 diabetes and microalbuminuria. Diabetes Care.

[19] Verzola D, Gandolfo MT, Gaetani G, Ferraris A, Mangerini R, Ferrario F (2008). Accelerated senescence in the kidneys of patients with type 2 diabetic nephropathy. Am J Physiol Renal Physiol.

[20] Gadalla SM, Cawthon R, Giri N, Alter BP, Savage SA (2010). Telomere length in blood, buccal cells, and fibroblasts from patients with inherited bone marrow failure syndromes. Aging.

